# Robust presurgical functional MRI at 7 T using response consistency

**DOI:** 10.1002/hbm.23582

**Published:** 2017-03-21

**Authors:** Pedro Lima Cardoso, Florian Ph. S. Fischmeister, Barbara Dymerska, Alexander Geißler, Moritz Wurnig, Siegfried Trattnig, Roland Beisteiner, Simon Daniel Robinson

**Affiliations:** ^1^ High Field Magnetic Resonance Centre, Department of Biomedical Imaging and Image‐guided Therapy Medical University of Vienna Lazarettgasse 14, A‐1090 Vienna Austria; ^2^ Study Group Clinical fMRI, Department of Neurology Medical University of Vienna Währinger Gürtel 18‐20, A‐1090 Vienna Austria

**Keywords:** fMRI analysis, presurgical planning, ultra‐high field, modified BOLD response, UNBIASED

## Abstract

Functional MRI is valuable in presurgical planning due to its non‐invasive nature, repeatability, and broad availability. Using ultra‐high field MRI increases the specificity and sensitivity, increasing the localization reliability and reducing scan time. Ideally, fMRI analysis for this application should identify unreliable runs and work even if the patient deviates from the prescribed task timing or if there are changes to the hemodynamic response due to pathology. In this study, a model‐free analysis method—UNBIASED—based on the consistency of fMRI responses over runs was applied, to ultra‐high field fMRI localizations of the hand area. Ten patients with brain tumors and epilepsy underwent 7 Tesla fMRI with multiple runs of a hand motor task in a block design. FMRI data were analyzed with the proposed approach (UNBIASED) and the conventional General Linear Model (GLM) approach. UNBIASED correctly identified and excluded fMRI runs that contained little or no activation. Generally, less motion artifact contamination was present in UNBIASED than in GLM results. Some cortical regions were identified as activated in UNBIASED but not GLM results. These were confirmed to show reproducible delayed or transient activation, which was time‐locked to the task. UNBIASED is a robust approach to generating activation maps without the need for assumptions about response timing or shape. In presurgical planning, UNBIASED can complement model‐based methods to aid surgeons in making prudent choices about optimal surgical access and resection margins for each patient, even if the hemodynamic response is modified by pathology. *Hum Brain Mapp 38:3163–3174, 2017*. © **2017 The Authors Human Brain Mapping Published by Wiley Periodicals, Inc.**

## INTRODUCTION

Presurgical mapping aims to identify essential functional brain regions which are close to the lesioned tissue to be excised. The process allows the best surgical approach to be determined and allows the neurosurgeon to plan the resection extent and identify post‐operatory risks to the patient. The “gold standard” cortical mapping technique is Direct Cortical Stimulation (DCS), which is performed during awake craniotomy. As well as being invasive, this approach presents an additional risk of cortical damage and seizures to the patient. In contrast, functional MRI (fMRI) provides a repeatable, non‐invasive means of localizing essential cortical regions. Used in a presurgical planning context, this technique may allow the extent of the resection to be increased, surgery time to be reduced and may improve preservation of function after surgery [Petrella et al., 2006]. Functional regions close to the pathology are commonly localized via the acquisition of a number of fMRI runs, in which a task is executed that generates activation relating to the function of interest; most commonly motor, language, and memory. The General Linear Model (GLM) [Friston et al., 1995] is the most common approach to generating activation maps in fMRI. The GLM needs the task processing periods to be known reliably, and incorporates a number of assumptions about the shape and timing of the blood oxygenation level‐dependent (BOLD) signal changes in response to an impulse stimulus—the Hemodynamic Response Function (HRF). In many contexts it is difficult to record if and when a task was performed. In addition, a number of exceptions to GLM assumptions about the shape and timing of the HRF have been documented, particularly with modified neurovascular coupling encountered close to tumors. These potential confounds motivate the use of analysis methods that do not share the GLM's assumptions about when tasks are executed or the shape and timing of the HRF.

Patients frequently have problems responding promptly to cues, causing deviation in the timing of the execution. Task processing may also occur at unanticipated times or with unexpected duration, with some tasks comprising a number of mental processes which cannot be monitored externally (e.g., the “hometown‐walking” task [Beisteiner et al., 2008; Jokeit et al., 2001; Roland and Friberg, 1985]). In practice, even for simple sensory tasks and stimuli, the clinic may not have access to MR‐compatible equipment capable of recording all responses. Thus, performance is often monitored visually, which introduces error into the estimation of the stimulus timing [Baudendistel et al., 1996].

In addition to difficulties assessing task processing periods, it is difficult to accurately predict the BOLD response. This may likewise confound model‐based analysis approaches. The HRF differs in shape and time‐to‐peak between brain regions and participants [Handwerker et al., 2004]. BOLD signal changes may be negative [Raichle et al., 2001; Shulman et al., 1997] or transient [Uludag, 2008], and the response shape varies across the brain [Gonzalez‐Castillo et al., 2012]. Inter‐subject and inter‐regional variability in the shape of the hemodynamic response can reduce BOLD sensitivity even in healthy populations [Handwerker et al., 2004]. Developmental differences [Arichi et al., 2012; Colonnese et al., 2008] and consumption of vasoactive substances can also alter the temporal course of the BOLD response [Liau et al., 2008]. Neurovascular uncoupling has been reported in both low grade [Zaca et al., 2014] and high grade brain tumors, preventing activation detection [Hou et al., 2006]. Furthermore, the time‐to‐peak of the HRF may be modified in regions of pathology [Wang et al., 2012] and the concentration of deoxyhemoglobin may exhibit atypical behavior [Fujiwara et al., 2004]. Cerebrovascular diseases and arteriovenous malformations (AVMs) have also been reported to originate reduced BOLD response in the ipsilesional motor cortex [Carusone et al., 2002], and decreased regional cerebral blood flow (rCBF) and perfusion [Fiehler et al., 2009], respectively, modifying the HRF. This introduces variability in activation patterns among brain tumor patients [Holodny et al., 2000] and a potential inability to detect viable neuronal tissue [Ulmer et al., 2004].

Even in healthy populations, sustained negative [Raichle et al., 2001; Shulman et al., 1997], phasic [Harms et al., 2005] and transient [Uludag, 2008] cerebral blood flow (CBF) and BOLD responses have been reported, and interest in the variability and reproducibility of BOLD responses to multiple repetitions of simple tasks has reemerged [Gonzalez‐Castillo et al., 2012]. In their totality, these studies constitute a reliable body of evidence that reproducible, non‐model‐conform responses occur in a wide range of contexts.

Patients are less likely to be able to adhere to prescribed timing and suffer modification to the HRF as a result of neuropathologies—particularly in the regions close to the tumor which are of primary interest. The described confounds are therefore particularly pertinent in fMRI for presurgical planning. Model‐free analysis methods represent an attractive solution, as they do not require assumptions about task timing, the shape of the HRF, or linearity in the response. Independent Component Analysis (ICA) [Beckmann, 2012], for instance, has been shown to have considerable advantages over the GLM in the context of presurgical planning at ultra‐high field (UHF) [Robinson et al., 2013]. This method has not been widely embraced in the clinical context due to the need to assess and interpret the large number of components (ICs) generated. The Finite Impulse Response (FIR) approach [Goutte et al., 2000], like ICA, makes no assumptions about the shape of the HRF. It consists instead of a flexible model which comprises a set of consecutive impulses (the FIR basis) that together span the ON/OFF period. Despite its inherent insensitivity to consistent shifts in response timing and modifications of the HRF shape, this method has not, to our knowledge, been applied in presurgical fMRI.

Preoperative fMRI paradigms often consist of repeated executions (runs) of blocks of identical tasks and timing [Stippich et al., 2015]. Splitting the total fMRI period into several runs allows patients short breaks and enables the reliability of activation to be assessed [e.g., Beisteiner et al., 2000]. With the clinically established “risk map” approach in mind [Beisteiner et al., 2000], Stevens et al. have recently shown that the reliability of fMRI presurgical mapping may be improved via optimization of preprocessing pipelines using a multi‐run acquisition scheme [Stevens et al., 2016]. Our own group has recently introduced a model‐free fMRI analysis method (UNBIASED) which makes use of the information in multiple runs to derive a measure of response consistency [Cardoso et al., 2016]. This is an extension of the BIASLESS method of Levin and Uftring [2001], in which voxel time courses are correlated between runs—an approach which shares some similarities with the interparticipant correlation (IPC) method of Hejnar et al. [2007]. We demonstrated UNBIASED's sensitivity to non‐model‐conform responses, and results in healthy volunteers were shown to be, in general, less contaminated by false positive results than the GLM. Additionally, the method was shown to be robust to consistent delays, altered HRF, reproducible anomalous responses, and able to accurately depict and discard low quality runs from the analysis [Cardoso et al., 2016]. The features of this approach make it well suited to application in presurgical clinical fMRI.

Presurgical planning generally benefits from acquisition of fMRI data at UHF strength with associated increase in time series SNR, BOLD sensitivity [Beisteiner et al., 2011, 2013; Geissler et al., 2014; Trattnig et al., 2015, 2016], and specificity to BOLD signal fluctuations in the microvasculature [Zhang et al., 2009]. These factors lead to the possibility of reducing measurement time and improving the reliability with which activation may be detected. In anticipation of the approval of the regulatory authorities for the use of 7 Tesla (T) MRI scanners for diagnostic purposes, we explore the clinical potential of 7 T fMRI for presurgical planning using UNBIASED analysis. The simplicity, specificity, and sensitivity of the approach has the potential to allow unreliable fMRI runs to be identified at the time of measurement, reducing the likelihood of the need for repeated localization sessions and allowing the measurement to be terminated when reliable results have been achieved.

Multi‐run 7 T fMRI data were acquired in a group of patients with brain tumors and epilepsy and analyzed with UNBIASED and the GLM. We assess the ability of UNBIASED to identify unreliable runs, generate maps of reliability of activation response and identify non‐model‐conform responses with a view to improving the clinical potential of UHF fMRI.

## MATERIALS AND METHODS

### Participants

Ten patients (mean age 29 ± 15 years old, 4 females) took part in this 7 T study. Most patients were being considered for surgery to excise tumors or epileptogenic foci (see Table 1 for patient demographics and clinical details). Presurgical localization for those patients was performed at 3 T, and is not described here. All patients were in a good general state of health at the time of measurement. All patients but one (P9) had no reported motor deficits. P9 suffered from dystonia of the upper extremities. Nevertheless, all patients could move the hand whose cortical representation was closest to the pathology against resistance, and were able to perform the task.

**Table 1:** Patient demographics and measurement details

Patient ID	Age	Gender	Head coil (# elements)	Number of runs completed	Pathology
P1	36	M	8	8	Right frontal tumor, unknown origin
P2	32	F	24	8	Temporal lobe resection left (status post glioblastoma)
P3	14	M	24	10	Cryptogenic epilepsy of the right parietal lobe
P4	21	M	24	8	Right central tumor, unknown origin
P5	28	M	24	8	Oligodendroglioma II, frontal lobe right
P6	21	F	32	8	Temporal lobe epilepsy right, status post partial temporal lobe resection right
P7	13	M	32	8	Extra‐temporal epilepsy
P8	53	F	32	8	Left parietal tumor, unknown origin
P9	21	M	24	7	Fibrillary astrocytoma (grade II), temporal lobe epilepsy right
P10	55	F	24	7	Left precentral tumor, unknown origin

Patient IDs begin with “P” and are used in other images and descriptions in the text.

This study was approved by the Ethics Committee of the Medical University of Vienna and all adult participants provided written informed consent prior to inclusion. In the case of minors this was provided by their legal guardians.

### Task Description

Patients were asked to perform up to 10 runs of a hand paradigm in a block design consisting of self‐paced repetitive opening and closing of the hand whose cortical representation was closest to the pathology with a frequency of approximately 1 Hz. Many patients performed a smaller number of runs due to reduced tolerance (see Table 1). Each run comprised four rest and three movement phases of 20 s each, presented in an ABABABA design (A: rest phase; B: task phase). Commands to commence and cease movement were communicated via the scanner speaker system during image acquisition.

### Data Acquisition

Images were acquired with a 7 T Siemens MAGNETOM scanner (Siemens Healthcare, Erlangen, Germany). Three different RF head coils were used, as hardware upgrades were undertaken during this study. The coils were an 8‐channel coil (Rapid Biomedical, Würzburg, Germany), a 24‐channel coil (Nova Medical, Wilmington, MA, USA), and a 32‐channel coil (Nova Medical). Table 1 lists which coil was used for each patient measurement. To minimize head movement, individually cast plaster helmets were used [Edward et al., 2000].

Functional images were collected with a 2D single‐shot gradient echo EPI sequence, with 34 slices oriented parallel to the Anterior Commissure–Posterior Commissure (AC–PC) plane, matrix size of 128 × 128, FOV = 230 × 230 mm (nominal 1.8 × 1.8 × 3.0 mm in‐plane resolution, 0.3 mm gap), TE/TR = 22/2,500 ms, flip angle of 80°, partial Fourier encoding of 3/4, receiver bandwidth of 1,446 Hz/pixel, and parallel imaging with a GRAPPA factor of 2. Two dummy excitations were used before the acquisition of 57 volumes per run and the first volume was discarded to achieve quasi‐equilibrium in longitudinal magnetization.

### fMRI Analysis

#### Data preprocessing

Image preprocessing was carried out in general accordance with the approach used by the Clinical fMRI Study Group at the Department of Neurology of the Medical University of Vienna for presurgical mapping [Beisteiner et al., 2010, 2011; Fischmeister et al., 2013; Foki et al., 2007]. Preprocessing was carried out in the native space of the EPI with FSL [Smith et al., 2004], with the exception of baseline correction in UNBIASED. The fMRI time series were slice timing corrected with SLICETIMER and motion corrected using MCFLIRT [Jenkinson et al., 2012], with 6 degrees of freedom. Each run was registered to the first volume of the middle run using FLIRT [Jenkinson et al., 2002], with 12 degrees of freedom. Voxel‐wise low frequency baseline signal drift was removed from the fMRI time series. In UNBIASED, this was achieved via second‐order polynomial regression (performed in MATLAB), a preprocessing step equivalent to high pass filtering (HPF) in the GLM. For the GLM, default HPF with a cut‐off frequency of 1/128 Hz was used. No normalization or spatial smoothing was performed for both analyses.

#### GLM analysis

The GLM approach as implemented in SPM8 (http://www.fil.ion.ucl.ac.uk/spm) was used to analyze the patient data and provide a point of comparison with UNBIASED. A canonical HRF was used, with no model derivatives or motion correction regressors. Only positive *t*‐values were considered.

#### UNBIASED analysis

UNBIASED was applied to functional localization of the primary motor cortex in the same group of patients. The method's ability to automatically identify and exclude compromised runs was assessed and regions of modified response shape were investigated.

**Figure 1:**
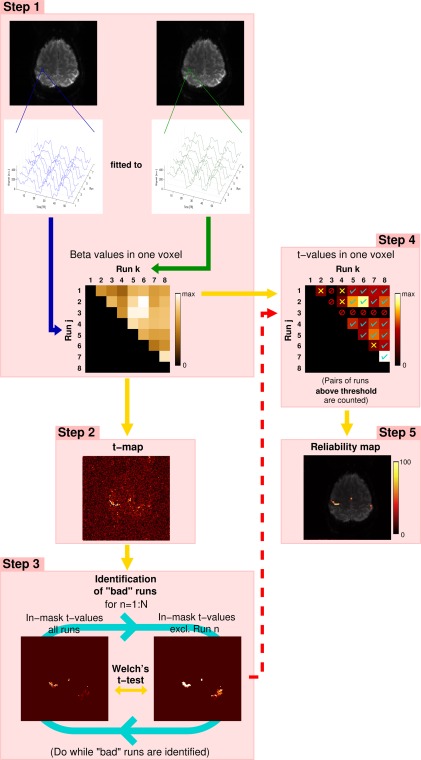
The main steps in UNBIASED, illustrated for 8 runs of a hand task presented in an ABABABA block design, (A: rest phase; B: task phase). Voxel‐wise fit (beta) values are calculated between the time courses of all non‐identical combinations of runs for each voxel. Time courses for a single voxel in a region activated by the task are shown (Step 1). Step 2: For each voxel, *t*‐values are calculated from the beta values of all non‐identical combinations of runs. Step 3: “Bad” runs are identified by performing a Welch's *t*‐test between the *t*‐map derived from all runs and that which excludes the run under consideration (Run *n*). Run 3 is excluded in this example (red “forbidden” signs in Step 4). Voxel‐wise *t*‐values are thresholded at an uncorrected *P* < 0.001. Those *t*‐values exceeding this threshold are counted (cyan ticks in Step 4). Those that fail to fulfill this criterion are marked with yellow crosses (Step 4). From all the “good” pairs of runs, the proportion of supra‐threshold *t*‐values to the total (in %) is used to generate the reliability map (Step 5)—the final result in UNBIASED. [Color figure can be viewed at http://wileyonlinelibrary.com]

In contrast to the GLM, the “model” in UNBIASED is unique for every voxel: it is the time course in the same voxel in a different run. The steps in UNBIASED are graphically illustrated in Figure 1. In brief, they comprise:

- Voxel‐wise calculation of beta values (Step 1) and *t*‐values (Step 2) from fits between time courses from non‐identical runs;
- Identification of “bad” runs via statistical comparison of *t‐*maps obtained with the inclusion and exclusion of each run (Step 3); and
- Assessment of how consistently each voxel is activated (Step 4) yielding the final activation measure (“Reliability map”; Step 5).

#### Comparison of GLM and UNBIASED results

Activation maps calculated with the GLM and UNBIASED were visually inspected for primary motor cortex activation. The level of artifact contamination in the results was additionally assessed by visual comparison between essential motor cortex activation [e.g., primary motor area (M1), somatosensory cortex, and supplementary motor area (SMA)] and regions such as the edge of the brain, white matter regions, and high contrast interfaces (e.g., CSF vs. gray matter) where false positive activation elicited by task correlated motion generally manifests.

### Assessment of UNBIASED Features

#### Identification of “bad” runs

The effectiveness of the identification of compromised runs in UNBIASED was assessed by reference to the GLM results for individual runs. Additionally, subject‐level UNBIASED results generated with and without the exclusion of the runs identified as “bad” were compared by calculating the percentage change in reliability values in a generous ROI (drawn by hand) containing activation in the primary motor area.

#### Regions of modified response shape

It has been shown that UNBIASED provides improved detection of consistent non‐model‐conform responses [Cardoso et al., 2016]. To allow these to be identified in the patient group in this study, the quality of fits between the model and the data were assessed for UNBIASED and an in‐house implementation of the GLM (ihGLM) using data preprocessed identically (i.e., including baseline correction). The model is the canonical HRF in the GLM and the time course in a different run in UNBIASED. The ihGLM implementation was necessary for the calculation of the fit residuals required in the computation of the quality of fits. Voxel‐wise goodness of fit was assessed using coefficients of determination calculated for both methods. These were used to compute the ratio $r_{UG}$ [as defined in Cardoso et al., 2016]—a measure of disparity between the goodness of fit of UNBIASED and the GLM. In this measure, positive values indicate better fit quality for UNBIASED, negative values for the GLM. Clusters containing contiguous voxels with a better goodness of fit in either the GLM (negative range) or UNBIASED (positive range) were selected and investigated to examine if the voxel time courses corresponded to the canonical HRF or not. This was achieved via visual comparison between average time courses (and standard deviations) computed in cubic ROIs with size 5 × 5 × 5 voxels positioned at around the center of these clusters and the GLM regressor, constructed by convolving the canonical HRF with the block design response timing (boxcar function).

The effect of the inclusion of compromised runs and of habituation of the response on the sensitivity of the GLM, UNBIASED, and Finite Impulse Response (FIR) methods is reported in Supporting Information.

## RESULTS

### Comparison of GLM and UNBIASED Results

**Figure 2:**
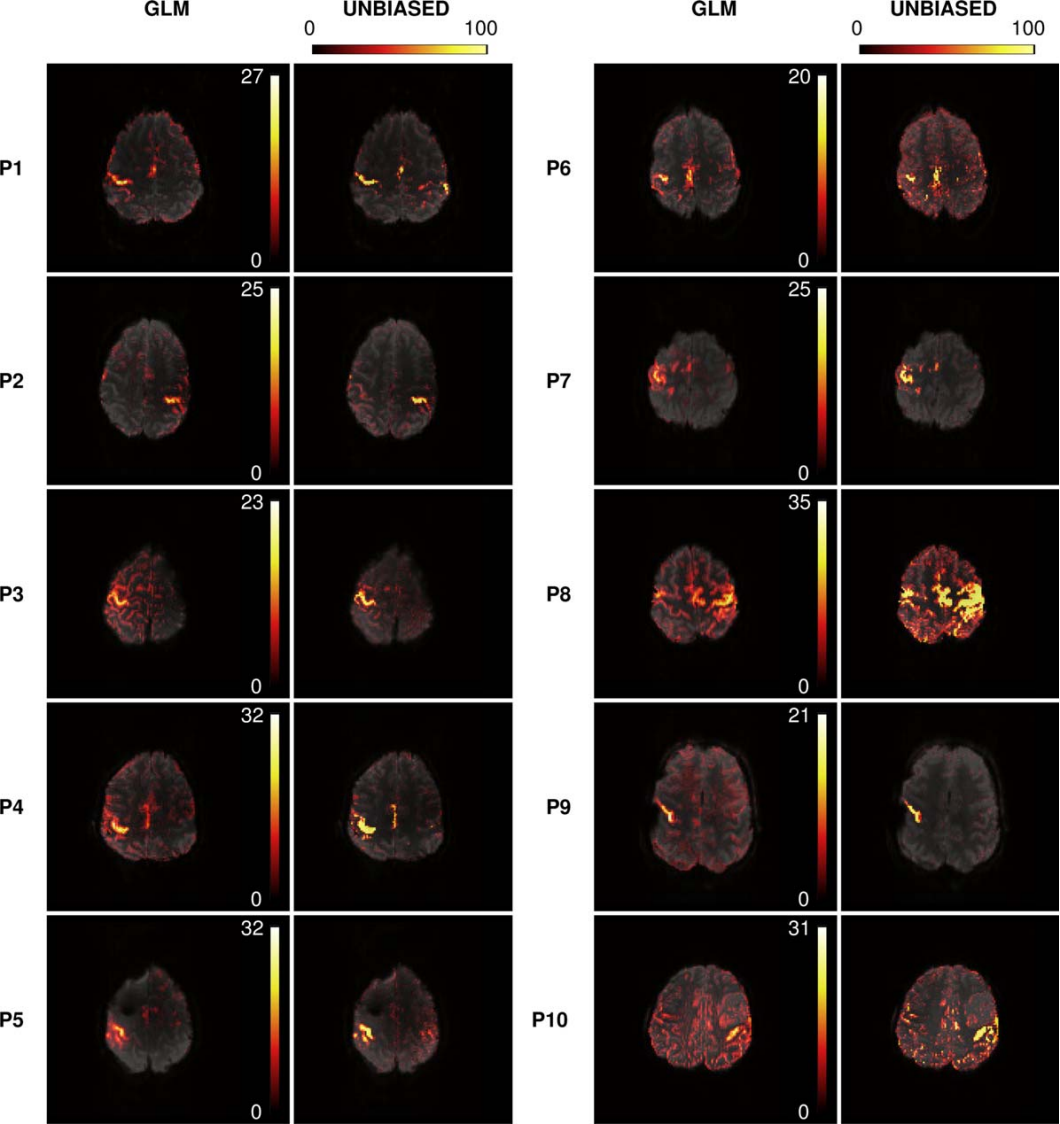
A comparison of hand motor localizations in 10 patients using GLM *t*‐maps (positive *t*‐values) [1st and 3rd columns] and unthresholded UNBIASED reliability maps [2nd and 4th columns]. Motor activation was detected with both methods for all patients. UNBIASED results generally suffered less from artifact contamination. Images are presented with a transparency of 25% in radiological convention. [Color figure can be viewed at http://wileyonlinelibrary.com]

Unthresholded UNBIASED results and positive GLM *t*‐values are shown in Figure 2. It was possible to identify the primary representation of the motor cortex in UNBIASED results in all patients. These localizations were in agreement with the GLM results. UNBIASED results suffered from less artifact contamination than those from the GLM in most cases (see P1, P2, P3, P4, P7, P9, and P10 in Fig. 2). In the remaining cases (P5, P6, and P8), spurious activations were observed. These corresponded to regions with time courses which were negative with respect to the canonical HRF, consistent across runs, and coincided with negative *t*‐value activations present in the GLM maps (not shown).

### UNBIASED Features

#### Identification of “bad” runs

**Figure 3:**
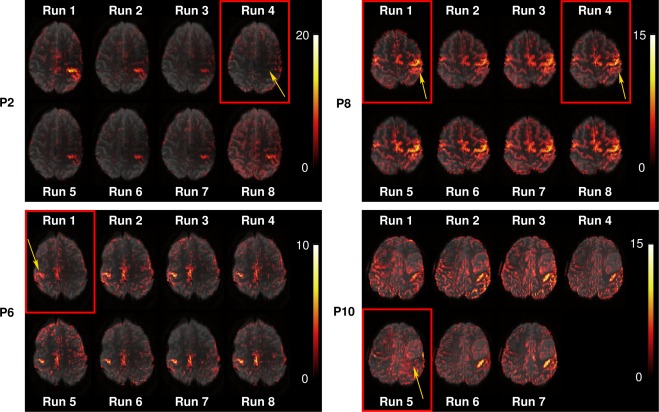
GLM results (positive *t*‐values) for four patients in which runs with low quality results were identified by UNBIASED. Activation was absent in patients P2, Run 4 and P10, Run 5 and weak in patients P6, Run 1 and P8, Run 1 and 4. Images are presented with a transparency of 25% in radiological convention. [Color figure can be viewed at http://wileyonlinelibrary.com]

UNBIASED identified compromised runs in four out of ten patients' results: P2 (Run 4), P6 (Run 1), P8 (Run 1 and 4), and P10 (Run 5). GLM analysis of individual runs in these patients (Fig. 3) shows no activation (P2 and P10; Fig. 3, at yellow arrows) or weak activation (P6 and P8) in the runs identified as “bad” by UNBIASED, and better results in other runs. For the four patients in whose data compromised runs were identified, an average increase of 19.9% ± 14.2% was observed in the UNBIASED reliability values following exclusion of these runs from the analysis.

#### Regions of modified response shape

**Figure 4:**
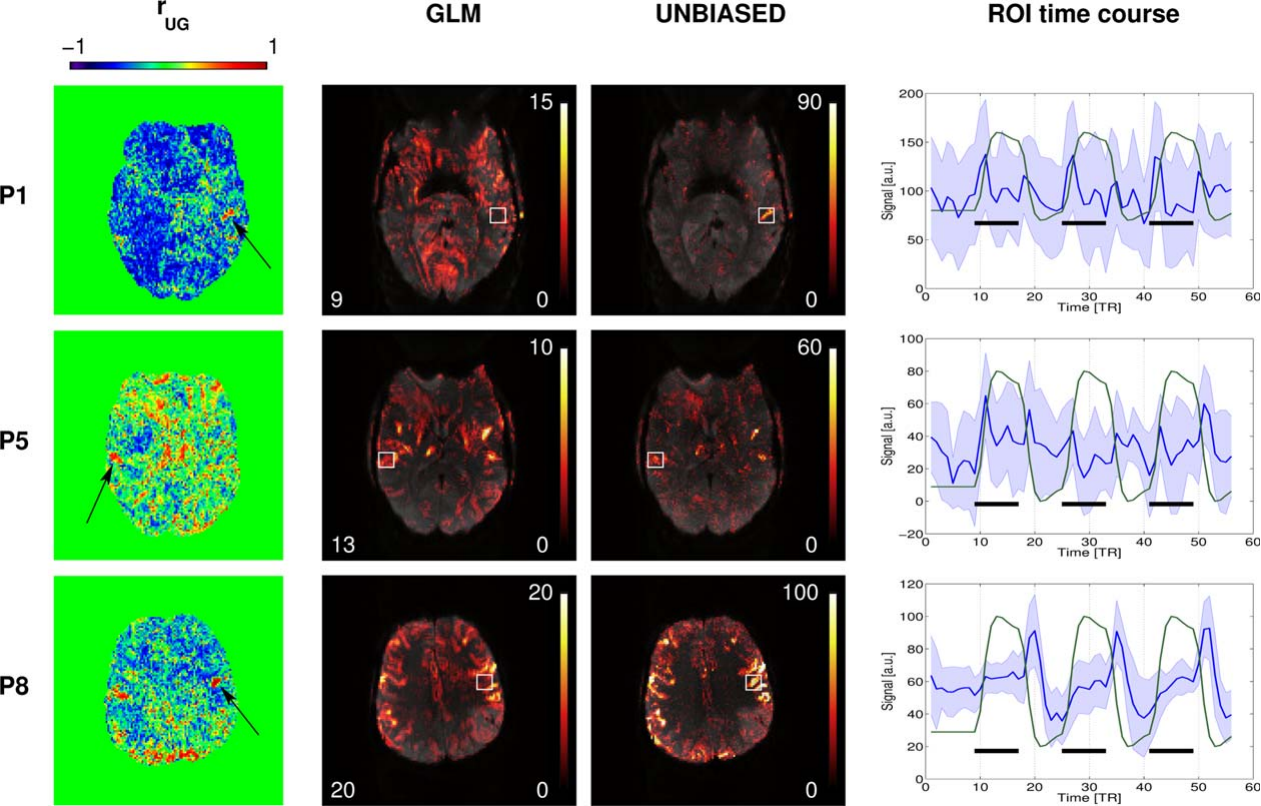
*Left column*: Maps of $r_{UG}$ for three patients who had regions with modified response shapes and discrepancies between GLM and UNBIASED results. *Black arrows* point to clusters of $r_{UG}>0$ (better quality of fit in UNBIASED). *Middle columns*: GLM (*left*) and UNBIASED (*right*) activation and reliability maps in radiological convention (25% transparency). *Right column*: Plots of the mean time courses (*blue line*) and standard deviation (*blue shaded area*) in cubic ROIs (*white boxes*) centered on the contralesional (*top*) and ipsilesional (*middle and bottom*) sides of the motor cortex for these patients. *Green lines* represent the GLM model regressor and *black bars* the stimulus timing. [Color figure can be viewed at http://wileyonlinelibrary.com]

Primary motor regions with reproducibly modified response shape were identified in three patients (P1, P5, and P8). In addition to the sustained smooth response present in a large extent of the dorsal region of the motor cortex (see Fig. 2), temporal signal changes time‐locked with the task were observed in ventral cortical areas, both in the ipsilesional hemisphere (P5—secondary somatosensory cortex, and P8—primary motor cortex) and the contralesional hemisphere (P1—superior temporal sulcus), as illustrated in Figure 4. Average time courses extracted from cubic ROIs centered on these regions reveal transient signal changes that occurred primarily during task‐switching periods (i.e., at the start and/or end of the task block). These non‐model‐conform responses resulted in poorer fit quality and thus reduced BOLD sensitivity in the GLM. The sensitivity of UNBIASED was uncompromised in those regions, with reliability values in the approximate range 50%–80%. Broad areas with better goodness of fit in the GLM were observed [e.g., P1, Fig. 4 (blue regions)] that generally corresponded to false positives containing time courses that were not reproducible over runs and, therefore, identified as having low response reproducibility in UNBIASED.

## DISCUSSION

In this study, we applied a model‐free analysis method, UNBIASED, to presurgical planning fMRI at ultra‐high field. UNBIASED integrates information from a number of runs with identical timing, using the signal change in each voxel of each run as a predictor for the signal change in the same voxel in other runs. This allows innate quality assurance—identifying low quality runs—and provides a measure of how consistently each voxel is activated by the task performed. Since it is not based on assumptions about the response shape and/or duration, UNBIASED is sensitive to activation‐related signal changes even if there are deviations from task timing and non‐model‐conform BOLD responses.

Multi‐run fMRI acquisitions allow patients short breaks between runs, which facilitates cooperation and reduces the likelihood of motion during acquisition. Generally, the responsible clinician determines the number of fMRI runs to be acquired on the basis of how challenging the detection of activation is in the affected cortical area and patient compliance. However, even if the number of runs is as low as two (the minimum required in UNBIASED), the method was shown to accurately depict activation [Cardoso et al., 2016].

UNBIASED offers automated data quality assurance via identification of runs that compromise the quality of the subject‐level analysis either because of technical artifacts (e.g., motion, drift, slice timing error, ghosting, …) or poor performance. This is particularly desirable in clinical fMRI, as the activation maps need to be available shortly after the examination for therapeutic decision‐making purposes. This study confirms that such runs, which inevitable occur in a clinical context, are correctly identified.

Gonzalez‐Castillo et al. [2012] reported modified response shape and magnitude time‐locked with the task extending beyond areas of primary relationship to the task in healthy volunteers. Using divergent task paradigms, topographically dissociated regions displaying both sustained and transient responses have been reported, with transient responses being generally associated with attention, detection of novelty, or task switching regions [Fox et al., 2005a; Konishi et al., 2001]. Alterations in transient responses in clinical populations have additionally been reported using a working memory task [Fox et al., 2005b]—a paradigm relevant in presurgical fMRI for the identification of functional cortical landmarks responsible for memory processing. Therefore, interregional differences in the temporal characteristics of the response may potentially emphasize differences in the role of these regions with respect to the task [Formisano and Goebel, 2003; Smolders et al., 2007], broadening knowledge of the dynamics of the functional organization of both the healthy and diseased brain. Until the clinical interpretation of these transient responses has been fully explored it seems wise to adopt the precautionary principle and ensure that the tissue concerned be spared. In our patient cohort, UNBIASED was able to detect activation regions with consistently modified response shape and time‐locked to the task that were not present in GLM results. Factors that potentially affect the HRF parameters have been observed in neuropathological conditions. In brain tumors, a decrease in amplitude [Hou et al., 2006; Zaca et al., 2014] and a delay in onset of the HRF have been reported [Wang et al., 2012]. Similar findings were observed in cerebrovascular diseases due to changes in vascular reactivity [Carusone et al., 2002]. Brain arteriovenous malformations (AVMs) represent a typical condition where regional cerebral blood flow (rCBF) and perfusion is significantly reduced [Fiehler et al., 2009], modifying the HRF. UNBIASED's sensitivity to consistent response shape and timing effects suggests that robust results may be achieved regardless of atypical hemodynamics. UNBIASED is expected to be equally sensitive to activation in newly‐formed functional areas which arise due to neuroplastic reorganization associated with the pathology. Even if the response shape in these regions differs from that in the corresponding primary functional areas, it is presumed to be consistent across runs.

Other model‐free methods have been applied to ultra‐high field presurgical planning data. ICA has been used to identify non‐model‐conform activation foci and separate activation from artifacts, for instance [Robinson et al., 2013]. The disadvantage with ICA is that it generates a large number of components which need to be assessed by the experimenter. Components may be ranked by the likelihood that they are task‐related, based on spatial and temporal features (e.g., correlation between frequency spectra of model time courses and frequency spectra of ICs) [Robinson et al., 2013]. However, the similarity between the frequency spectra of activation and stimulus‐correlated motion components impedes a simple identification of components of interest. Spatial features, on the other hand, require further analysis and/or co‐registration of the functional data to standard brain templates, which can be challenging, particularly in the clinical setting. Finally, task related ICs may also be split in two or more components, further complicating the process.

Localization uncertainties are critical, particularly in presurgical planning. A measure based on reliability is of utmost importance. Using a model‐based analysis, Beisteiner et al. [2000] showed that a combination of a voxel reliability measure (from multiple runs) with a high correlation threshold contribute to a reduction in false positive and false negative activation. Similarly, UNBIASED integrates information from multiple runs to originate a measure of reliability. This may aid the surgeon in the decision about resection margins or necessity of alternative treatment strategies as it could point out the degree of involvement a particular area possesses in task execution.

The age range of the participating patients in this study varies from 13 to 55 years old (see Table 1). These were compliant and able to perform the task. Results are expected to be consistent also in younger or older populations that are able to perform the task. If partial or total paresis of the extremities would impede the patient to perform the task, a somatosensory stimuli task (e.g., touch, pressure, flutter, and vibration/movement) could, as well, replace the task paradigm for central sulcus identification, in which case the somatosensory cortex located in the post‐central gyrus would be activated instead.

Three different head coils were used in this study, as data was acquired while hardware upgrades were being performed. This could have had impacted the image SNR and/or introduced higher GRAPPA noise with regards to an acquisition with a coil with a larger number of elements. This, however, does not seem to have hindered the identification of essential motor cortex in the patient for which the 8 channel coil was used (P1). Precise and nearly artifact‐free identification of the motor cortex was attained in this case.

The main limitations of UNBIASED are that the fMRI experiment must be constructed as a number of runs with identical timing, and that it is incapable of distinguishing activation from different tasks or contrasting conditions [Cardoso et al., 2016]. In its current implementation, UNBIASED is not able to differentiate between activation and deactivation. This may, however, not be a disadvantage in the context of presurgical planning in which the main goal is to identify essential functional landmarks (e.g., the central sulcus for the sensorimotor region) which are recruited by task execution, irrespective to the sign or shape of the response. A possibility to remove this limitation could be, for instance, the implementation of a hybrid approach, in which information about the sign of the response would be obtained from the conventional approach (e.g., from the beta (fit) values in the GLM) and used in UNBIASED or to calculate the correlation between the boxcar function corresponding to the stimulus timing (accounting for a reasonable hemodynamic delay) and the BOLD response in each voxel. This could aid applications where knowledge about the sign of the response may be desirable (e.g., in cognitive neurosciences). Even though the patient data in this study was subject to an unusually small amount of motion because individual plaster head casts were used, UNBIASED showed similar or lower sensitivity to motion than the GLM with the paradigm used. It is also insensitive to artifact sources such as motion uncorrelated with the task, as well as non‐task, sporadic, and/or physiological activations if these are not consistent across runs. This is generally a useful feature [Cardoso et al., 2016] but does mean that UNBIASED would not be an ideal method to assess activation in cognitive tasks comprising distinct processing phases which are variable in duration (such as mathematical problems). However, the sensitivity of model‐based methods is also reduced if the responses do not correspond to the chosen model shape and timing. In such cases, model‐independent methods such as Independent Component Analysis may be more appropriate.

High reliability values could, in principle, arise from physiological fluctuations if the acquisition of the fMRI runs were temporally synchronous with some physiological cycle (e.g., cardiac or respiratory). No coherent relationship exists, in general, between physiological cycles across runs [Cardoso et al., 2016] but some subjects have been shown to demonstrate task‐correlated breathing when performing cognitive or emotional tasks [Birn et al., 2009; Huijbers et al., 2014], effectively adapting their breathing to the presentation cycle. This has not, to our knowledge, been reported in motor tasks. If this effect is identified (e.g., by correlating physiological measures and the stimulus time course) and has the potential to interfere with the interpretation of fMRI results it can be mitigated using Retroicor [Glover et al., 2000], Nuisance Variable Regression [Lund et al., 2006] or ICA [Tohka et al., 2008] to remove physiological components and reduce respiration effects [Birn et al., 2006]. Finally we would note that the GLM is also affected by stimulus‐correlated motion and breathing if the paradigm is composed of quite long blocks (as opposed to rapid events, which allow motion and activation to be separated to some extent [Birn et al., 1999]).

## CONCLUSION

Application of the model‐free analysis method UNBIASED to ultra‐high field presurgical fMRI resulted in reliable activation maps of the primary representation of the motor cortex. Unreliable runs were identified automatically and UNBIASED functional maps suffered from less artifact contamination than those from a GLM analysis. Additionally, cortical regions exhibiting consistently modified response shape and timing could be identified with UNBIASED but not with the GLM.

The ability to identify consistent but atypical BOLD responses is a valuable feature when compromised task performance or modified HRF are expected. This makes it particularly attractive as a complementary approach for presurgical planning, aiding clinicians in decisions concerning surgical approach, and necessity and extent of therapeutic resection of impaired brain tissue.

## DISCLOSURE

The authors report no conflict of interest concerning the materials or methods used in this study or the findings specified in this article.

## ETHICS APPROVAL

Ethics Committee of the Medical University of Vienna, Austria.

## Supporting information

Supporting Information
