# Supplementary material for: Robust presurgical functional MRI at 7 T using response consistency
Source: Hum Brain Mapp. 2017 Mar 21;38(6):3163–74. doi: 10.1002/hbm.23582 (PMC5434844; doi:10.1002/hbm.23582)
Supplement: Supplementary file 1 — Supporting Information [file HBM-38-3163-s001.docx]

**Supporting material to the article: “Robust presurgical functional MRI at 7 T using response consistency”**

The Finite Impulse Response (FIR) approach is a potentially useful analysis method in the context of presurgical fMRI. It shares some characteristics of UNBIASED, such as an insensitivity to consistent shifts in the timing of the response. It does not, however, provide an intrinsic means of identifying “bad” runs, and retains the assumption that the response is similar in each block (in contrast to UNBIASED, in which the assumption is of similar responses across runs). The influence of “bad” runs on the sensitivity of FIR was investigated for the four patients in which UNBIASED identified and excluded compromised runs (P2, P6, P8, and P10).

Habituation is one of the simplest and most common learning mechanisms. It manifests as a decrement in behavioral and neuronal response to a stimulus after repeated presentations (Rankin, et al., 2009; Thompson, 2009) and has been observed in various brain regions (Klingner, et al., 2011; Mutschler, et al., 2010; Plichta, et al., 2014) including the motor cortex (Dirnberger, et al., 2004). The effect of response habituation (both within blocks and between blocks) on GLM, FIR, and UNBIASED results was modeled using simulated data in which the effect of including a range of numbers of compromised runs was also explored.

**Supporting methods**

*Functional FIR maps in patients with compromised runs*

Functional runs for those patients with runs identified as “bad” in UNBIASED were analyzed with an FIR approach. This analysis was implemented in SPM8 by constructing 16 regressors (one per time point of the ON/OFF periods). Each regressor consisted of tent functions with the timing of the stimulus onsets, moving across regressors in a temporal window that spanned 16 volumes (see **Sup. Fig. 1**). The regressors were not convolved with the canonical HRF or any other basis function. The F contrast used to produce the final activation map was constructed as a squared identity matrix with the size of the number of regressors (16×16) replicated as many times as the corresponding number of runs performed by each patient.

| 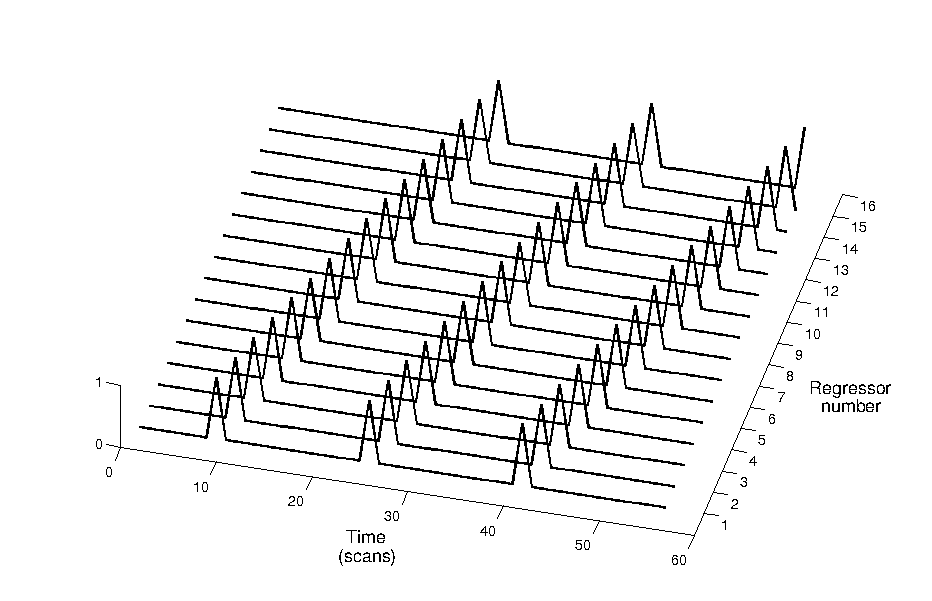 |
| --- |
| **Sup. Fig. 1**  Regressors used in the FIR analysis have the shape of a tent function and span a time window of 16 volumes (i.e. the period of one ON/OFF block). |

*Response shape and the effect of bad runs on GLM, UNBIASED, and FIR functional maps*

Eight runs of simulated data were generated by replicating, for each run, one volume of P1’s first fMRI run 56 times (the number of time points in the paradigm used in this study). Gaussian-distributed white noise was added to the time courses of each voxel using the MATLAB function “awgn” with a signal-to-noise ratio of 30 dB. Four spherical regions with a radius of 4.5 voxels (8.1 mm in-plane) were defined (regions A-D in **Sup. Fig. 3** (top left)). A different response shape was added to each region in addition to the noise (**Sup. Fig. 3** (top right)). These were:

A) A boxcar function convolved with the canonical HRF;

B) A boxcar function with habituation between blocks, convolved with the canonical HRF;

C) A boxcar function with habituation within each block, convolved with the canonical HRF;

D) A boxcar function with habituation between blocks and within each block, convolved with the canonical HRF.

All block design boxcar functions corresponded to the timing used in this study. Habituation across the blocks in each run was simulated by reducing the height of the second and third ON blocks (of the boxcar function) to 75% and 50% of the height of the first one, respectively. Habituation within blocks was generated by exponentially reducing the height within the ON blocks to 70% of the value at its onset. Habituation between blocks and within each block (D) was generated with a combination of both aforementioned habituations. The same responses were added to each run.

The simulated runs were analyzed with the GLM, UNBIASED, and FIR. To investigate the effect of the presence of compromised runs, the analysis was repeated in datasets with an increasing number of “bad” runs. This was achieved by incrementally replacing good runs (up to a maximum of 4) by a run containing only noise.

**Supporting results**

*Functional FIR maps in patients with compromised runs*

FIR activation maps for the patients with compromised runs identified with UNBIASED are illustrated in **Sup. Fig. 2**. These were - with the proportion of “bad” runs to the total indicated in brackets - P2 (1/8), P6 (1/8), P8 (2/8), and P10 (1/7). Despite the presence of one or two “bad” runs, the FIR approach was able to identify the hand area of the primary motor cortex. The effect of a larger proportion of poor runs is investigated in the simulated data.

| 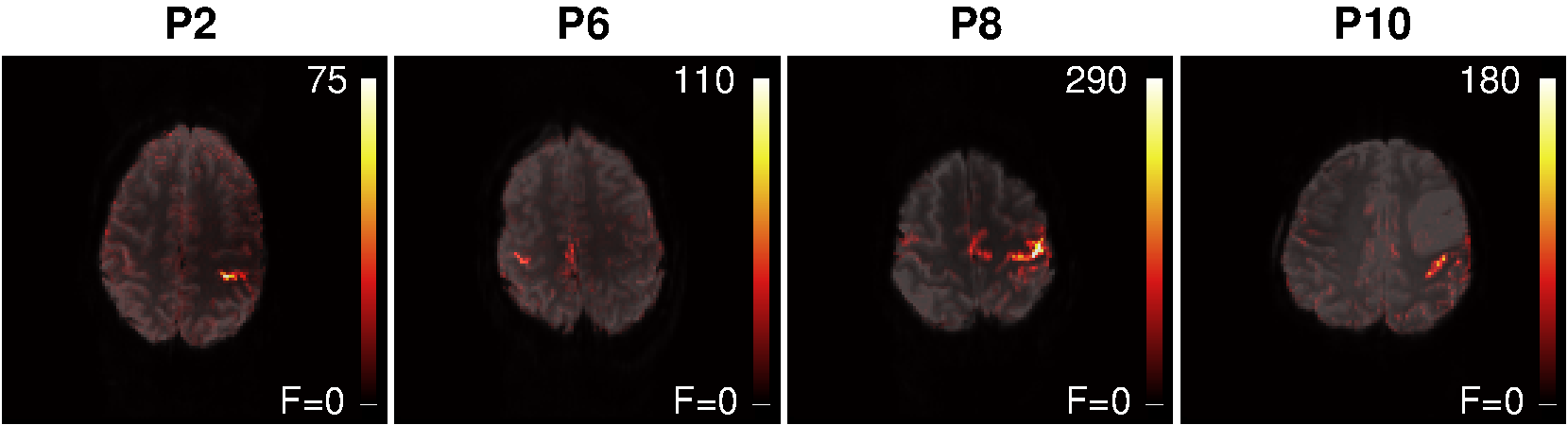 |
| --- |
| **Sup. Fig. 2**  Finite Impulse Response activation maps calculated in four patients in which runs were identified as “bad” with UNBIASED. |

*Response shape and the effect of bad runs on GLM, UNBIASED, and FIR functional maps*

A comparison of the effect of the shape of the response and the presence of compromised runs in the GLM, UNBIASED, and FIR results is illustrated in **Sup. Fig. 3** (bottom) for different response shapes and number of “bad” runs included. The GLM and FIR showed reduced sensitivity where there was habituation either between blocks and/or within block (regions B-D) compared to the canonical response (with no habituation) in region A. UNBIASED was largely unaffected by changes to the response shapes. In region D, a small reduction of the response reliability was observed with regards to the other regions. In the presence of an increasing number of compromised runs, the GLM suffered from a decrease in sensitivity in all regions. The FIR was affected to an even larger degree. UNBIASED shows consistent reliability values with an increasing number of “bad” runs due to its intrinsic capability of excluding these from the calculation of the reliability of the response.

| 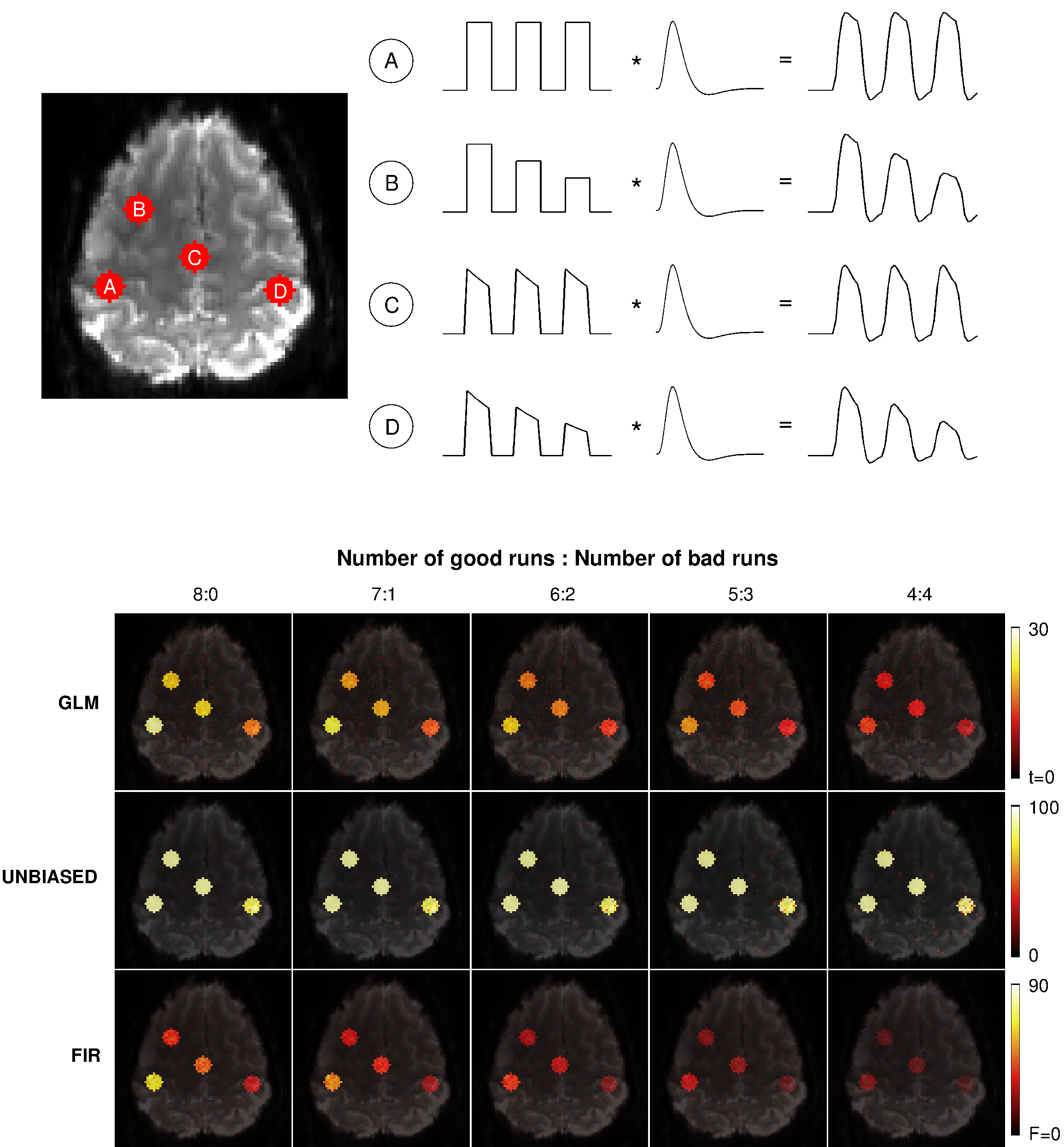 |
| --- |
| **Sup. Fig. 3**  *Top:* Localization and shape of the time courses added to the simulated data. All block design boxcar functions (A-D) correspond to the timing used in this study and were convolved with the canonical HRF. The time series differ in the type of habituation introduced (A: no habituation; B: habituation between blocks; C: habituation within ON blocks; D: habituation between blocks and within each block).  *Bottom:* Effect of the shape of the response and presence of compromised runs on GLM, UNBIASED, and FIR results. Images are presented with a transparency of 25%. |

**Supporting Discussion:**

Analysis of the fMRI data from the patients with compromised runs with FIR yielded precise identification of the central sulcus, the functional landmark for the primary motor cortex. This approach was robust to the presence of one or two “bad” runs in *in vivo* data. In contrast, a strong decrease in sensitivity was observed in the simulated data, particularly when there was no response in more than two runs (**Sup. Fig. 3**). This may have resulted from the fact that even though the excluded runs had been identified as “bad” in UNBIASED, the patients were nonetheless performing the task. However, the data quality may have been strongly affected by noise or motion in these runs, yielding to the reduced activation observed with respect to the remaining good runs (**Fig. 3**), leading to their exclusion with UNBIASED.

**References:**

Dirnberger, G., Duregger, C., Lindinger, G., Lang, W. (2004) Habituation in a simple repetitive motor task: a study with movement-related cortical potentials. Clinical Neurophysiology, 115:378-384.

Klingner, C.M., Nenadic, I., Hasler, C., Brodoehl, S., Witte, O.W. (2011) Habituation within the somatosensory processing hierarchy. Behavioural Brain Research, 225:432-436.

Mutschler, I., Wieckhorst, B., Speck, O., Schulze-Bonhage, A., Hennig, J., Seifritz, E., Ball, T. (2010) Time Scales of Auditory Habituation in the Amygdala and Cerebral Cortex. Cerebral Cortex, 20:2531-2539.

Plichta, M.M., Grimm, O., Morgen, K., Mier, D., Sauer, C., Haddad, L., Tost, H., Esslinger, C., Kirsch, P., Schwarz, A.J., Meyer-Lindenberg, A. (2014) Amygdala habituation: A reliable fMRI phenotype. Neuroimage, 103:383-390.

Rankin, C.H., Abrams, T., Barry, R.J., Bhatnagar, S., Clayton, D.F., Colombo, J., Coppola, G., Geyer, M.A., Glanzman, D.L., Marsland, S., McSweeney, F.K., Wilson, D.A., Wu, C.F., Thompson, R.F. (2009) Habituation revisited: An updated and revised description of the behavioral characteristics of habituation. Neurobiology of learning and memory, 92:135-138.

Thompson, R.F. (2009) Habituation: A history. Neurobiology of learning and memory, 92:127-134.
